# Reproducibility of semi-quantitative assessment of aortic valve calcification and valve motion on echocardiography: a small-scale study

**DOI:** 10.1186/s44156-024-00050-3

**Published:** 2024-07-01

**Authors:** D. Balian, B. Koethe, S. Mohanty, Y. Daaboul, S. H. Mahrokhian, J. Frankel, J. Li, A. Kherlopian, B. C. Downey, B. Wessler

**Affiliations:** 1 Tufts Medical Center, Boston, USA; 2 Harrison Memorial Hospital, Cynthiana, KY, USA; 3 Loma Linda University Health, Loma Linda, CA USA

**Keywords:** Echocardiography, Aortic stenosis, Semi-quantitative, Reproducibility, Inter-reader Reliability

## Abstract

**Background:**

Aortic stenosis (AS) is the most common degenerative valve disease in high income countries. While hemodynamic metrics are commonly used to assess severity of stenosis, they are impacted by loading conditions and stroke volume and are often discordant. Anatomic valve assessments such as aortic valve calcification (AVC) and valve motion (VM) during transthoracic echocardiography (TTE) can offer clues to disease severity. The reliability of these semi-quantitatively assessed anatomic imaging parameters is unknown.

**Methods:**

This is a retrospective study of semi-quantitative assessment of AVC and valve VM on TTE. TTEs representing a range of AS severities were identified. The degree of calcification of the aortic valve and the degree of restricted VM were assessed in standard fashion. AVC scores and valve motion were assessed by readers with varied training levels blinded to the severity of AS. Correlation and inter-reader reliability between readers were assessed.

**Results:**

420 assessments (210 each for AVC and VM) were collected for 35 TTEs. Correlation of AVC for imaging trainees (fellows and students, respectively), ranged from 0.49 (95% CI 0.18–0.70) to 0.62 (95% CI 0.36–0.79) and 0.58 (95% CI 0.30–0.76) to 0.54 (95% CI 0.25–0.74) for VM. Correlation of anatomic assessments between echocardiographer-assigned AVC grades was *r* = 0.76 (95% CI 0.57–0.87)). The correlation between echocardiographer-assigned assessment of VM was *r* = 0.73 (95% CI 0.53–0.86), *p* < 0.00001 for both. For echocardiographer AVC assessment, weighted kappa was 0.52 (0.32–0.72), valve motion weighted kappa was 0.60 (0.42–0.78).

**Conclusion:**

There was good inter-reader correlation between TTE-based semi-quantitative assessment of AVC and VM when assessed by board certified echocardiographers. There was modest inter-reader reliability of semi-quantitative assessments of AVC and VM between board certified echocardiographers. Inter-reader correlation and reliability between imaging trainees was lower. More reliable methods to assess TTE based anatomic assessments are needed in order to accurately track disease progression.

**Clinical Trial Number:**

STUDY00003100.

**Supplementary Information:**

The online version contains supplementary material available at 10.1186/s44156-024-00050-3.

## Introduction

Aortic stenosis (AS) is the most common valve disease in those aged 75 and older and progresses through fibrotic and calcific disease stages that ultimately lead to restricted valve motion (VM), obstruction of blood flow, remodeling of the left ventricle, heart failure, and death if left untreated [1]. While valve hemodynamics are the primary imaging features on transthoracic echocardiogram (TTE) used to assess valve disease severity, there is increasing interest in anatomic assessments of valve dysfunction [2]. Valve calcification and restricted VM observed during TTE are hallmark features of this disease and adjunctive imaging features that can be used to help stage disease severity. These features have also been proposed as tools to aid with risk stratification and prognosis [3, 4]. The reliability and reproducibility of semi-quantitative assessments of valve calcification and valve motion on TTE are unknown [2, 5]. 

Aortic valve calcification (AVC) and restricted VM are features of degenerative AS that can be seen on TTE and ultimately contribute to progressive pressure overload on the left ventricle. AVC progresses at variable rates and has demonstrated strong associations with traditional cardiovascular risk factors [5, 6]. This calcification coupled with pro-fibrotic processes ultimately lead to fusion of valve leaflets and restricted VM [7]. These phases demonstrate variable progression with women showing lower rates of calcification for any given stage of disease [8]. Semi-quantitative assessments of aortic valve anatomy are routinely reported and have been used in studies of AS disease progression [2, 9, 10]. Regardless of the quantification technique used, the baseline load of calcium on the aortic valve may be the most well-validated marker regarding the severity and progression of AS [2]. 

AVC as assessed by computerized tomography (CT) is the most accurate, reproducible, and well-validated marker of AS severity (and disease progression) at later disease stages, and is helpful in the diagnostic evaluation of patients with discordant echocardiographic markers of AS [2]. The reliability of semi-quantitative AVC and VM assessments during TTE imaging is unknown and remains a critical question since these assessments are incorporated into disease staging guidelines [11]. Highly reliable and reproducible TTE imaging would be preferable to CT due to ease of access, low cost of imaging, and lack of ionizing radiation. Here we conduct a study to assess the reproducibility and inter-reader reliability of standard semi-quantitative assessments of TTE-based AVC and VM assessments across the spectrum of AS between individuals of different levels of training.

## Materials and methods

### TTE selection protocol

This was a retrospective single center study of TTEs representing the full spectrum of AS. The study population was identified using a Phillips® ISCV search tool. This EMR-based search tool was used to identify patients with AS. Imaging from patients with ‘none, mild, moderate, or severe’ AS as classified by a board-certified echocardiographer (cardiologist with additional fellowship in cardiac imaging) were obtained and de-identified and randomly selected [11]. Patients were excluded if image quality was poor, either due to patient characteristics or quality of the study. For this study, each included study was independently reviewed by a board-certified echocardiographer to confirm severity of AS and to confirm that the imaging views were evaluable. Patients with prosthetic valves and inadequate aortic valve visualization were excluded.

### Image selection protocol

For each study, de-identified videos representing zoomed parasternal long axis (PLAX) and parasternal short axis at the level of the aortic valve (PSAX AoV) were used. During the course of this study these imaging views were displayed side by side for each reader. Imaging was displayed in a random order and readers were blinded to the reference AS severity label.

### Image interpretation protocol

Images were individually reviewed by 2 cardiologists boarded in echocardiography, 2 cardiology fellows, and 2 medical students. The cardiology fellows are post-graduate years 4–6. The medical students receive basic ultrasound training in their pre-clinical education and received an additional didactic session regarding PLAX and PSAX views of the aortic valve with various stages of AV disease. Readers were asked to grade AVC in standard fashion using a scale from 1 to 4 (1, no calcification; 2, mild was defined as few areas of dense echogenicity with little acoustic shadowing; 3, moderate as multiple larger areas of dense echogenicity; and 4, severe as extensive thickening and increased echogenicity with a prominent acoustic shadow). Degree of VM was graded from 1 to 4 (1, normal motion; 2, mildly restricted motion; 3, moderately restricted motion; and 4, heavily restricted motion).

### Statistical analysis

Reproducibility and inter-reader reliability were assessed. Reproducibility is defined as variation of the same measurement made on the same subject by different readers, while inter-reader reliability is defined as the ability for different readers to come to similar conclusions when shown the same image [10]. The correlation for AVC and VM grades assigned by readers with the same level of training was assessed using Spearman correlation with 95% confidence intervals. Inter-reader agreement was assessed using the Kappa statistic to compare agreement between two readers of the same training level. P-values of < 0.05 were considered statistically significant. Statistical analysis was performed using SAS 9.4 statistical software. Kappa ranges correspond to: ≤ 0 as indicating no agreement and 0.01–0.20 as none to slight, 0.21–0.40 as fair, 0.41– 0.60 as moderate, 0.61–0.80 as substantial, and 0.81–1.00 as almost perfect agreement [10]. 

## Results

This study included imaging from 35 patients represented by 70 videos (PLAX and PSAX of AoV), 2 for each patient. 6 readers analyzed AVC and valve motion from 35 TTEs (210 AVC labels and 210 valve motion labels). The imaging cohort included patients with no AS (*n* = 5), patients with mild AS (*n* = 10), patients with moderate AS (*n* = 10), and severe AS (*n* = 10). The echocardiographic characteristics of the patient images are shown in Supplemental Table 1. The median age was 73 years (IQR 13). 71% of the patients were men. 88% of the study population was white.

### AVC

Correlation between the echocardiographer-assigned AVC grades was *r* = 0.76 (95% CI 0.57–0.87 [*p* < 0.0001]) (supplemental Fig. 1). For fellows, correlation coefficients was *r* = 0.49 (95% CI 0.18–0.70 [*p* = 0.0027]) (supplemental Fig. 3). Correlation for medical students was *r* = 0.62 (95% CI 0.36–0.79 [*p* < 0.0001]) (supplemental Fig. 5). For inter-reader reliability between board certified echocardiologists, AVC weighted kappa = 0.52 (0.32–0.72). Inter-reader reliability for trainees ranged from 0.37 (0.14–0.61) to 0.5 (0.29–0.72).

### Valve motion

Correlation coefficients for echocardiographer-assigned assessment of VM was *r* = 0.73 (95% CI 0.53–0.86) [*p* < 0.00001]) (supplemental Fig. 2). For fellows, correlation coefficients were *r* = 0.58 (95% CI 0.30–0.76 [*p* = 0.0002]) (supplemental Fig. 4). The correlation between medical students were *r* = 0.54 (95% CI 0.25–0.74 [*p* = 0.0007]) (supplemental Fig. 6). For inter-reader reliability between board certified echocardiologists, VM weighted kappa = 0.60 (0.42–0.78). Inter-reader reliability for trainees ranged from was 0.29 (0.11–0.48) to 0.49 (0.27–0.72) for VM.

## Discussion

The main finding from this study is that there is good correlation for TTE-based assessments of AVC and VM though inter-reader reliability is modest. While anatomic assessments of valve morphology can be helpful in assigning AS grade and correlate with subsequent outcomes, standard TTE-based semi-quantitative assessments lack the precision and reproducibility needed to reliably track disease progression. More accurate assessment of AVC could provide better tools for assessing AS severity. These findings suggest the need for more accurate and reproducible TTE-based methods to assess aortic valve morphology.

TTE remains the primary imaging modality used to assess AS severity and its ability to assess both anatomic and hemodynamic changes associated with worsening AS makes it ideally suited for tracking disease progression [4, 11]. Contemporary clinical assessment of AS severity relies heavily on an integrative approach that combines a number of (mostly hemodynamic) imaging parameters in order to assign a summary severity grade. While there has been substantial interest in refining hemodynamic parameterization of this condition, these metrics are often discordant [9]. There has been less attention paid to the visual assessment of AVC or VM on TTE and a semi-quantitative approach to grading these metrics is commonly used in practice and has been integrated into imaging guidelines [11]. The data presented here suggest that the current approach may not yield reliable results.

There are some early efforts underway to improve the precision and reproducibility of TTE-based anatomic assessments of the aortic valve. In a previous single center study, a novel global calcium (GC) score, defined as the summed means of grayscale in 3 regions of interest (ROI) in PLAX and 5 ROI in PSAX was found to correlate with CT Agatston score [12]. Unfortunately, this analysis included only 14 patients with AS and to our knowledge has not been repeated. Investigators found similar success and application to echocardiographic measurement of GC in comparison to CT score [13, 14]. An investigation utilizing software that analyzes relative pixel brightness with the anechoic nature of blood as a control has been suggested as a potential tool for echocardiography experts to analyze AVC, finding a strong correlation between human expert and computer software assessment of calcium area for the same images [15]. 

More recently, a 2D-AVC ratio was defined as the average pixel density of the AV divided by the average pixel density of the aortic annulus was reported to correlate with hemodynamic severity of AS [16]. These results lay the groundwork for more accurate and reproducible assessments of AVC though remain limited in that they only use a diastolic PSAX image and do not integrate assessments of VM.

Restricted VM is an important anatomic feature of AS that has not yet been established as a prognostic variable in AS progression. As information on the sexual dimorphism of this condition emerges, and with evidence that calcification progresses at different rates (and to different thresholds) for men and women, a reliable and accurate method to assess VM could offer important insights about valve severity for those with fibrosis-dominant phenotypes. Additionally, as the age of artificial intelligence for echocardiography advances forward, attention to these morphologic features might improve confidence and agreement between providers when assessing severity of AS.

Semi-quantitative assessments in echocardiography are commonly used because quantitative analyses are time consuming and often discordant. As TTE workflows improve, higher reproducibility and accuracy should be expected. While automation tools continue to advance, they have yet to be trained on traditionally semi-quantitative measures. Adding these types of morphology assessments to the portfolio of automated measurements might ultimately help with more accurate and reproducible grading of AS, as well as other valve lesions [17]. This study included good quality TTE imaging and therefore represents a ‘best cases scenario’ for inter-reader correlation and reliability. In clinical practice, many factors impact the quality of TTE imaging, potentially worsening the real-world reliability of these parameters.

## Conclusions

There was good inter-reader correlation for semi-quantitative assessment of AVC and valve motion however inter-reader reliability was modest. More reliable methods to assess TTE based anatomic assessments are needed in order to accurately track disease progression.

### Electronic supplementary material

Below is the link to the electronic supplementary material.

Supplementary Material 1

Supplementary Material 2

## Data Availability

Data is not publicly available to protect patient privacy. Data is stored on encrypted hard drive on Tufts Medical Center servers.

## Abbreviations

AoV: Aortic Valve
AS: Aortic Stenosis
AVC: Aortic Valve Calcification
CT: Computerized tomography
GC: Global Calcium
PLAX: Parasternal Long Axis
PSAX: Parasternal Short Axis
ROI: Region of interest
TTE: Transthoracic Echo
VM: Valve Motion
