# Supplementary material for: Reproducibility of semi-quantitative assessment of aortic valve calcification and valve motion on echocardiography: a small-scale study
Source: Echo Res Pract. 2024 Jul 1;11:15. doi: 10.1186/s44156-024-00050-3 (PMC11215824; doi:10.1186/s44156-024-00050-3)
Supplement: Supplementary file 1 — Supplementary Material 1 [file 44156_2024_50_MOESM1_ESM.docx]

*Supplemental Figure 1: Board Certified Echocardiographers analysis of aortic valve calcification grade. r = 0.76 (95% CI 0.57-0.87). p < 0.0001. AVC: aortic valve calcification. Individual points are minimally displaced to show point density*

*Supplemental Figure 2: Board Certified Echocardiographers analysis of aortic valve motion. r = 0.73 (95% CI 0.53-0.86). p < 0.00001. VM: valve motion. Individual points are minimally displaced to show point density*

*Supplemental Figure 3: Cardiology Fellows analysis of aortic valve calcification grade. r = 0.49 (95% CI 0.18-0.70). p = 0.0027. AVC: aortic valve calcification. Individual points are minimally displaced to show point density*

*Supplemental Figure 4: Cardiology Fellows analysis of aortic valve motion. r = 0.58 (95% CI 0.30-0.76). p = 0.0002. VM: valve motion. Individual points are minimally displaced to show point density*

*Supplemental Figure 5: Medical Students analysis of aortic valve calcification grade. r = 0.62 (95% CI 0.36-0.79). p < 0.0001. AVC: aortic valve calcification. Individual points are minimally displaced to show point density*

*Supplemental Figure 2: Medical Students analysis of aortic valve motion. r = 0.54 (95% CI 0.25-0.74). p = 0.0007. VM: valve motion. Individual points are minimally displaced to show point density*
