# Supplementary material for: Reproducibility of semi-quantitative assessment of aortic valve calcification and valve motion on echocardiography: a small-scale study
Source: Echo Res Pract. 2024 Jul 1;11:15. doi: 10.1186/s44156-024-00050-3 (PMC11215824; doi:10.1186/s44156-024-00050-3)
Supplement: Supplementary file 2 — Supplementary Material 2 [file 44156_2024_50_MOESM2_ESM.docx]

|  | **Max AoV Gradient (mmHg)** | **Mean AoV Gradient (mmHg)** | **AoV Area (cm2)** | **Ao peak velocity (m/s)** | **SV LVOT (mL)** | **EF (%)** | **Systolic BP** | **Diastolic BP** |
| --- | --- | --- | --- | --- | --- | --- | --- | --- |
| No AS (5) | 7.4 | 3.58 | 2.5 | 1.31 | 65.5 | 54.0% | 145.4 | 86.8 |
| Mild AS (10) | 22.1 | 12.6 | 1.6 | 2.31 | 71.7 | 58.0% | 123.0 | 64.1 |
| Moderate AS (10) | 39.4 | 21.5 | 1.2 | 3.1 | 82.1 | 54.6% | 129.2 | 69.0 |
| Severe AS (10) | 66.4 | 38.5 | 0.8 | 4.0 | 74.5 | 62.0% | 140.0 | 74.0 |
| Total (35) | 37.6 | 21.3 | 1.4 | 2.9 | 76.5 | 58.0% | 136.0 | 76.8 |

Supplementary Table 1: Demographics of patient cohort (n = 35). All values are represented as averages. Cohort sizes are as follows: n=5 no AS, n = 10 for mild/mild-moderate AS, n = 10 moderate AS, n = 10 moderate-severe, severe AS. AoV: Aortic Valve, Ao: Aorta, SV: Stroke volume, LVOT: left ventricular outflow tract, EF: Ejection Fraction, BP: blood pressure
